# Artificial Intelligence in Public Health: Current Trends and Future Possibilities

**DOI:** 10.3390/ijerph191911907

**Published:** 2022-09-21

**Authors:** Daniele Giansanti

**Affiliations:** Centre Tisp, Istituto Superiore di Sanità, 00161 Rome, Italy; daniele.giansanti@iss.it; Tel.:+39-06-4990-2701

Artificial intelligence (AI) is a discipline that studies whether and how intelligent computer systems that can simulate the capacity and behaviour of human thought can be created.

Specific definitions can be obtained by focusing on applications and by measuring the reliability and effectiveness of behaviour after comparison with human behaviour [1].

What is expected from an AI-based system can be summarized in the following statements [1]:

1. It *must act* in a similar way to human beings: the product of the procedure performed by the artificial intelligent system should be indistinguishable from the one followed by a human being.
2. It *must think* in a similar way to human beings: the sequential activity that leads the intelligent system to face and solve a problem is comparable with the one followed by a human being.
3. It *must behave* (*act* and *think*) in a rational way: the method that leads the intelligent system to solve a problem is a formal structured process following the logic.
4. It *must obtain* the best possible result: the process that leads the intelligent system to solve the problem is the one that allows it to obtain the best-expected outcome based on the provided information.

AI has its roots in and includes scientific disciplines such as computational sciences and neural networks. Starting in the 1980s [2] we began to speak specifically of AI when Deep Blue-type system emerged and were found to be capable of dealing with the human beings in the game of chess and again later on, when NASA used dedicated applications such as Remote Agent to manage the activities of a spacecraft. From these first applications, it was clear that the use of AI has important implications for the user and on the environment; therefore, particular attention must be paid to the ethical, environmental, regulation, and social aspects of AI and to the need to increase transparency and responsibility in the process of use.

In some previous studies focused on AI in digital radiology and digital pathology we have seen this; that is, the real integration of AI in the *health domain* cannot be based on scientific development alone and cannot ignore ethical, regulatory, and social aspects, such as the acceptance of citizens and professionals [3,4,5,6].

In 2017, following a conference of world artificial intelligence experts promoted by the Future of Life Institute, a vademecum with 23 principles was drawn up, with broad consensus to address the ethical, social, cultural, and military issues of AI. The document was immediately signed by over 800 experts and later by thousands more [7].

In the *health domain*, when dealing with the health of the individual, all of this is particularly felt. This Special Issue, “Artificial Intelligence in Public Health: Current Trends and Future Possibilities” [8], aims to act as a collector among scholars on issues related to both the development and integration of AI in the health domain. This is addressed both with reference to current trends and by trying to grasp future developments.

This represents a new and hot research topic, as verified through a search on Pubmed, a reference database on issues related to biomedicine and the health domain.

If we search [9]:

*(Artificial Intelligence [Title/Abstract]) AND (current trends [Title/Abstract]) Sort by: Publication Date “artificial intelligence” [Title/Abstract] AND “current trends” [Title/Abstract]*

We can find 72 articles, 70 of which were published between 2018 and 2022.

Among these, we note a particular development of reviews, 46, all of which are recent (63.9%).

If we search [10]:

*(Artificial Intelligence [Title/Abstract]) AND (future possibilities [Title/Abstract]) Sort by: Publication Date “artificial intelligence” [Title/Abstract] AND “future possibilities” [Title/Abstract]*

We find 22 articles, all of which were published between 2018 and 2022.

Among these, we note a particular development of reviews, with 15 being found (68.2%).

The high percentage of reviews around these issues certainly denotes a great interest in the cultural and scientific mediation actions dedicated to the intensification of research in this area aimed both at the development and present and future integration of AI on the part of scholars. This is comforting and corroborates the idea of developing a Special Issue dedicated to this [8].

Much is expected of AI both today and tomorrow, both from a development point of view and via integration with the *health domain,* which must also pass regulations, ethical scrutiny, social acceptance.

Research in this area is strategic for the development of health systems and is inextricably linked to the development of digital health, both in regard to this collection, monitoring, and management of information and in regard to the management of hospital and connected government information systems. Think, for example, of the opportunities presented by wearable monitoring, big data, robotic assistance, rehabilitation, and surgery. The applications of artificial intelligence have received growing interest in many sectors, such as in those related to organ, functional tissue, and cell diagnostics [3,4,5,6]; care robotics, which assist in interventions, rehabilitation, and supporting the communication and assistance of disabled people [11,12,13,14]; the biomedicine sector, where it is implemented in applications from genetics to modelling [15,16,17,18]; and precision and personalized biomedicine [19,20,21,22,23,24]. The consolidation of technologies based on artificial intelligence in the *health domain* is intended to bring benefits to everyone, from the stakeholder to the patient, in the form of equity of care.

In the future, artificial intelligence is expected to have a strong impact on:

- The prevention of the onset of diseases in the individual and in society
- The provision of personal care and assistance.
- Society trends regarding diseases and the impact of biological and behavioural factors.
- The organization of hospital activities with regard to treatment, diagnostics, and decision-making processes.

Thanks to artificial intelligence, on the one hand, big data [25,26] will help us to predict diseases on an individual and collective basis and to identify and correct population behaviours; on the other hand, wearable technologies will allow us to monitor and collect individual medical information and to calibrate the care process. The integration of artificial intelligence with virtual reality and augmented reality [27,28] will allow us to create both virtual medicine services that citizens can access in a simple and direct way as well as robotic surgery applications that are increasingly effective and safe.

Many professionals will be involved in the process of developing and integrating AI into the *health domain* in the present as well as in the future. The sectors of research, diagnosis, and clinical therapy will be called upon to offer ideas and experts in this area. The government, educational, and political sectors will also be called upon to make important decisions. Therefore, stakeholders will have to take on important responsibilities.

The research sector, to which this editorial is addressed, will have a leading role since it is from this sector that not only algorithms and proposals for AI solutions but also concrete ideas of transfer to the *health domain* for a stable use in clinical/biomedical practice will emerge, accompanied by the actions of the regulators acting at all envisaged levels. This key role will also be assumed by the disciplines responsible for the certification of medical devices with artificial intelligence content, which will have to be harmonized and consolidated, particularly at the international level.

It is hoped that this Special Issue [8] will be useful for this purpose and that it will be capable of making important contributions in the levels of intervention related to these issues.
